# Neuro-Cognitive Effects of Acute Tyrosine Administration on Reactive and Proactive Response Inhibition in Healthy Older Adults

**DOI:** 10.1523/ENEURO.0035-17.2018

**Published:** 2018-04-30

**Authors:** Mirjam Bloemendaal, Monja Isabel Froböse, Joost Wegman, Bram Bastiaan Zandbelt, Ondine van de Rest, Roshan Cools, Esther Aarts

**Affiliations:** 1 Donders Institute for Brain, Cognition and Behaviour, Centre for Cognitive Neuroimaging, Radboud University, Nijmegen 6525EN, The Netherlands; 2 Department of Psychiatry, Radboud University Medical Center, Nijmegen 6500HB, The Netherlands; 3 Division of Human Nutrition, Wageningen University, Wageningen 6700AA, The Netherlands

**Keywords:** dopamine, functional MRI, healthy aging, response inhibition

## Abstract

The aging brain is characterized by altered dopamine signaling. The amino acid tyrosine, a catecholamine precursor, is known to improve cognitive performance in young adults, especially during high environmental demands. Tyrosine administration might also affect catecholamine transmission in the aging brain, thereby improving cognitive functioning. In healthy older adults, impairments have been demonstrated in two forms of response inhibition: reactive inhibition (outright stopping) and proactive inhibition (anticipatory response slowing) under high information load. However, no study has directly compared the effects of a catecholamine precursor on reactive and load-dependent proactive inhibition. In this study we explored the effects of tyrosine on reactive and proactive response inhibition and signal in dopaminergically innervated fronto-striatal regions. Depending on age, tyrosine might lead to beneficial or detrimental neurocognitive effects. We aimed to address these hypotheses in 24 healthy older human adults (aged 61–72 years) using fMRI in a double blind, counterbalanced, placebo-controlled, within-subject design. Across the group, tyrosine did not alter reactive or proactive inhibition behaviorally but did increase fronto-parietal proactive inhibition-related activation. When taking age into account, tyrosine affected proactive inhibition both behaviorally and neurally. Specifically, increasing age was associated with a greater detrimental effect of tyrosine compared with placebo on proactive slowing. Moreover, with increasing age, tyrosine decreased fronto-striatal and parietal proactive signal, which correlated positively with tyrosine’s effects on proactive slowing. Concluding, tyrosine negatively affected proactive response slowing and associated fronto-striatal activation in an age-dependent manner, highlighting the importance of catecholamines, perhaps particularly dopamine, for proactive response inhibition in older adults.

## Significance Statement

Healthy aging comes with altered dopamine functioning and is associated with reduced performance on cognitive control tasks, such as response inhibition. However, it is yet unclear whether reactive or proactive response inhibition is modulated by dopamine. We addressed this question by administering the catecholamine precursor tyrosine in a double blind, placebo-controlled, randomized intervention study. Tyrosine decreased proactive response slowing, not reactive stopping, as a function of increasing age. Concurrently, proactive fronto-striatal and parietal blood oxygen level-dependent (BOLD) signal decreased after tyrosine with increasing age. These findings, especially in striatum, demonstrate that proactive, rather than reactive response inhibition, is dopamine dependent. Moreover, tyrosine’s effect on brain and cognition became detrimental with increasing age, questioning the cognitive enhancing potential of tyrosine in healthy aging.

## Introduction

The aging brain is characterized by alterations in dopamine functioning (Kaasinen and Rinne, 2002; Braskie et al., 2008). Age-related decreases in dopamine receptor and transporter binding have been linked to impairments in cognitive functions such as attention, episodic and working memory (Bäckman et al., 2006) and age-related increases in dopamine synthesis capacity have been related to decreased neural reward processing (Dreher et al., 2008). In aged experimental animals, administration of a D1 receptor agonist improved working memory performance (Cai and Arnsten, 1997). Similarly, enhancing dopamine levels in humans with the drug L-Dopa (the direct dopamine precursor) improved age-related impairments in episodic memory performance and reinforcement learning (Chowdhury et al., 2012, 2013).

Tyrosine is a large non-essential neutral amino acid (LNAA), naturally present in food. Tyrosine is the precursor of the catecholamines, converted to dopamine via L-Dopa and the enzymes tyrosine hydroxylase (TH) and aromatic l-amino acid decarboxylase and to noradrenaline by dopamine β-hydroxylase (Molinoff and Axelrod, 1971). Research in rodents showed that orally administered tyrosine reaches the brain (Glaeser et al., 1983). Tyrosine administration increases dopamine metabolites in CSF, like homovanillic acid (HVA), in rats (Scally et al., 1977) and in patients with Parkinson’s disease (Growdon et al., 1982). In young adults, tyrosine administration improved cognitive control functions such as response inhibition, task switching, and working memory, especially in demanding circumstances (for review, see Deijen, 2005; Jongkees et al., 2015). In the aging brain, tyrosine may similarly improve cognitive functioning.

Aging is accompanied by deficits in inhibitory functions, both in terms of the inhibition of irrelevant information, e.g., sensory suppression (Gazzaley et al., 2008; Healey et al., 2008), as well as in terms of response inhibition, such as in stop-signal tasks (Kramer et al., 1994; Bedard et al., 2002; van de Laar et al., 2011). Two forms of response inhibition have been distinguished: reactive response inhibition is the process of canceling an ongoing response at the moment this is needed (i.e., outright stopping), whereas proactive response inhibition entails the preparation for stopping when this may become necessary, e.g., based on cues held in working memory. Age-related impairments have been shown in both reactive inhibition (measured with stop-signal reaction time; SSRT) and proactive inhibition (measured with anticipatory response slowing), particularly under high information load (i.e., high information processing demands for interpreting the stop-signal probability cues; Bloemendaal et al., 2016; Kleerekooper et al., 2016). It is unclear whether tyrosine-induced modulation of catecholaminergic signaling in older adults will affect reactive and/or proactive response inhibition. For reactive response inhibition, pharmacological animal and human genetic work shows both dopaminergic as well as noradrenergic involvement (Eagle et al., 2007; Congdon et al., 2009; Ghahremani et al., 2012; Rae et al., 2016; Schippers et al., 2016; see also Eagle and Baunez, 2010). Catecholaminergic modulation of proactive response inhibition has never been formally tested, but experimental animal work implicates dopamine in a variety of processes contributing to proactive response inhibition (Bari et al., 2009; Bari and Robbins, 2013). Further indirect evidence for a role of dopamine in proactive inhibition comes from neuroimaging studies. Specifically, midbrain signal was associated with stop-signal probability and RT adjustments (Boehler et al., 2011; Zandbelt et al., 2013).

In this neuro-imaging study, we investigated effects of acute, oral tyrosine administration on reactive and (load-dependent) proactive response inhibition and associated signal in dopamine-innervated fronto-striatal regions of the aging brain. We used a dose of 150 mg/kg body weight, in accordance with most previous studies in young volunteers (Shurtleff et al., 1994; Neri et al., 1995; Thomas et al., 1999; Magill et al., 2003; Mahoney et al., 2007), but see work by Colzato and colleagues for beneficial effects on cognition in young volunteers with much smaller doses (Colzato et al., 2013, 2014a, 2014b; Steenbergen et al., 2015). By investigating older adults, we could also assess the potentially beneficial effects of tyrosine in aging. We expected to find beneficial effects of tyrosine on brain and behavior across the group of older adults. Given our recent findings of age-related differences in the peripheral plasma response to oral tyrosine administration (van de Rest et al., 2017) and given differences in the effect of dopaminergic agents between younger and older adults (Turner et al., 2003), we also explored the possibility that tyrosine’s effects would vary as a function of age. Using a smaller age range, we do not expect generational differences to influence the effects of dopaminergic agents, such as differences in education or computer experience that would differ in a cross-sectional design with larger age differences. The oldest relative to the youngest older adults are presumably most dopamine deprived and, thus, may benefit most from administration of dopamine’s precursor tyrosine. However, whereas aging has been associated with reduced dopamine receptor and transporter binding, it has also been shown to be accompanied by upregulation of (dorsal) striatal dopamine synthesis capacity (Braskie et al., 2008; Berry et al., 2016). This upregulation of synthesis capacity has been related to, if anything, worse rather than better neurocognitive functioning relative to young adults (Dreher et al., 2008; Berry et al., 2016). Moreover, a recent study with multiple oral tyrosine doses (100, 150, and 200 mg/kg, but no placebo condition) showed decreased cognitive performance with increased tyrosine dose in older adults (van de Rest et al., 2017). Therefore, in the current placebo-controlled study, administration of a high dose (150 mg/kg) of the dopamine precursor tyrosine might also impair instead of improve neuro-cognitive function in the oldest adults, with presumably the greatest upregulated dopamine synthesis capacity.

## Materials and Methods

### Participants

Participants met the following criteria: aged between 60 and 75, right handed, functioning within normal limits of general cognitive function [mini-mental state examination (MMSE); Folstein et al., 1975; cutoff ≥27 of 30), no depression or anxiety [hospital anxiety and depression scale (HADS) score <11; Bjelland et al., 2002], an estimated verbal IQ > 85 (Schmand et al., 1991), not suffering from neurologic or psychiatric disorders, no first degree relatives suffering from schizophrenia, bipolar disorder, or major depressive disorder, no history of alcohol or drug abuse, no habitual smoking defined as less than a pack of cigarettes a week for the last year, current or past (within last 12 months) participation in a specific cognitive training program, no contraindications for MRI, no daily use of β blockers, no use of medication interfering with tyrosine’s action (monoamine oxidase inhibitors and other antidepressants, sympathomimetic amines, and opioids), no thyroid problems and no low-protein diet, no endocrine or metabolic disorders such as hepatic or renal problems, no repetitive strain injury (RSI) or sensorimotor handicaps, blindness, or colorblindness.

**Figure 1.**
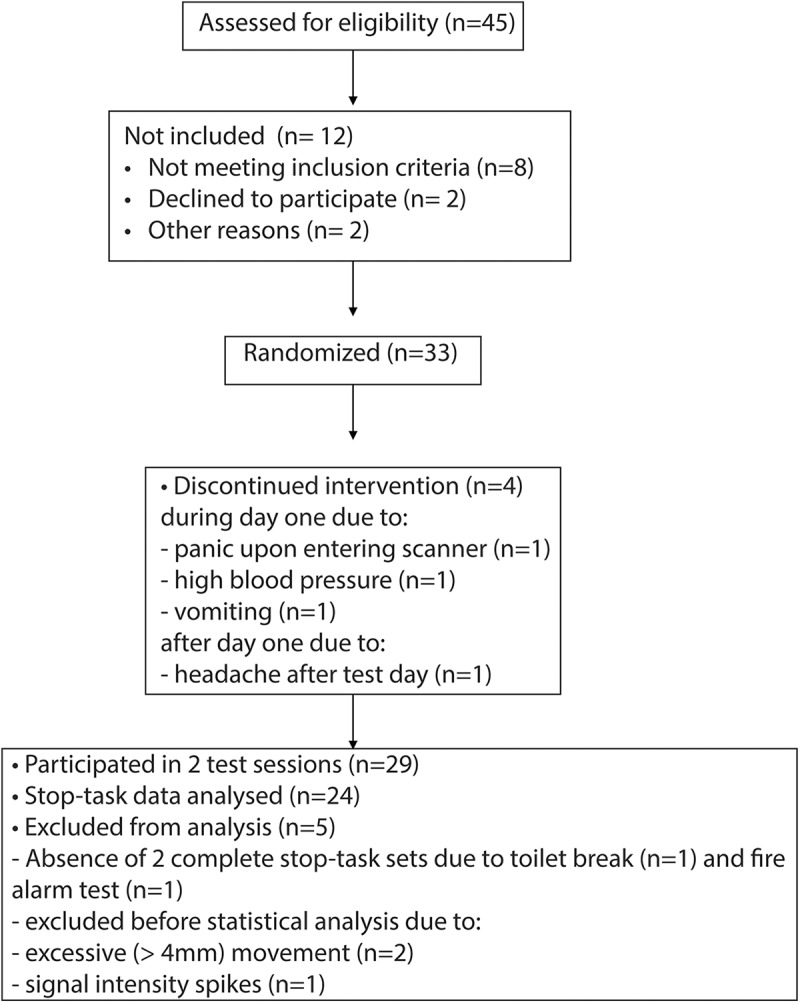
Flowchart of participants through the study.

Participants were recruited via adverts in local newspapers, websites, and associations for older adults. After informing potential participants about the inclusion criteria, 45 older adults were invited for a pre-screen session. After the pre-screen, we invited 33 participants for the test sessions (Fig. 1). Of these 33, 29 participants completed two test sessions. Of the four participants who did not complete all test sessions, three participants were excluded during test day 1 (due to panic on entering the scanner, high blood pressure, or vomiting) and one after test day 1 (due to headache after the test day). Of the 29 participants who completed both test days, a final sample of 24 healthy older adults were included in the analyses (mean age: 67.5, range 61–72, 15 men). Of the five participants who were not included in the analyses, two participants did not finish the stop-task on one of the sessions, and three participants were excluded before statistical data analysis: two due to excessive movement (>4-mm translation) and one due to signal intensity spikes.

The experiment was approved by the local ethics committee (CMO 2014-1172), and all participants gave written informed consent. The study was preregistered at the Dutch trial register (www.trialregister.nl) 159 under number NTR4938.

### Intervention

Our participants received a dosage of 150 mg/kg tyrosine or placebo, adjusted to body weight as determined during the pre-screen session (Procedure). The European Food Safety Authority determined in July 2011 that tyrosine is proven to contribute to the normal synthesis of catecholamines (EFSA Panel on Dietetic Products Nutrition and Allergies, 2011). In accordance with most previous studies in young volunteers (Shurtleff et al., 1994; Neri et al., 1995; Thomas et al., 1999; Magill et al., 2003; Mahoney et al., 2007; but see Colzato et al., 2013, 2014a, 2014b; Steenbergen et al., 2015), we used a dosage of 150-mg tyrosine or placebo per kilogram of body weight. For reference, a daily required intake of phenylalanine and tyrosine for adults was estimated at 14 mg/kg/d (World Health Organization, 1985) or 39 mg/kg/d in a more recent study (Basile-Filho et al., 1998).

The placebo product was a mixture of 54 mg/kg dextrine-maltose (i.e., carbohydrates; product name Fantomalt by Nutricia) with maizena (110 mg/kg, ratio Fantomalt/cornstarch = ∼1/2). The ratio of Fantomalt to cornstarch was adjusted such that the placebo and tyrosine mixture have an equal energy value, similar structure and aftertaste. Equal taste experience for tyrosine and placebo was ensured in a formal sensory experiment by a specialized dietician from the Division of Human Nutrition of Wageningen University (E.Siebelink).

The tyrosine and placebo product were mixed with a carrier: banana-flavored yoghurt (Arla Food Nederland). Weighing of the doses and preparing and coding the samples was performed by a staff member who was not further involved in the study.

### Physiologic and mood measurements

To monitor wellbeing, participants completed mood ratings, assessing calmness, contentness, and alertness (Bond and Lader, 1974). Moreover, we assessed levels of the catecholamine metabolites [HVA, vanillylmandelic acid (VMA), 3-methoxy-4-hydroxyphenylglycol (MOPEG), and 3,4-dihydroxyphenylacetic acid (DOPAC) in urine to measure peripheral effects.

### Procedure

All participants were tested between November 2014 and August 2015 at the Donders Center for Cognitive Neuroimaging, Nijmegen, The Netherlands. Participants were pre-screened in a separate 4-h session and trained on the tasks. During this pre-screen session, participants signed informed consent, were screened on all the in- and exclusion criteria, and completed several neuropsychological measures: verbal IQ as measured with the Dutch version of the NART (NLV; Schmand et al., 1991), HADS (Bjelland et al., 2002), and the Barratt impulsiveness scale (BIS-11; Patton et al., 1995; Table 1). Moreover, participants were trained on the three tasks they were going to perform during the test sessions and were weighed to determine the individual tyrosine dose.

**Table 1.** Trait demographics and neuropsychological tests

Variable	
Age (years)	67.5 (0.6)
Sex (women/men)	W: 9; M: 15
Verbal IQ	114.5 (2.0)
HADS total	3.5 (0.5)
HADS anxiety	2.3 (0.4)
HADS depression	1.2 (0.3)
MMSE	29.1 (0.3)
BIS-11 motor	20.9 (0.6)
BIS-11 cognitive	14.8 (0.6)
BIS-11 non-planning	21.7 (0.7)
BIS-11 total	57.4 (1.1)

Data represent mean (SEM) except for the variable sex, for which data reflect frequencies. Verbal IQ is defined by scores on the Dutch version of the NART, MMSE, BIS-11, subscales and total score. Men and women are equally distributed across the whole group.

On both test sessions (of at least one week apart), the same procedure was followed except for the supplement taken: tyrosine or placebo, counterbalanced across participants.

**Figure 2.**
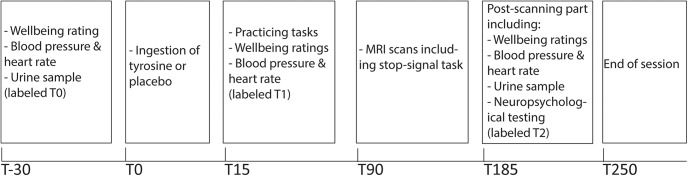
Schematic of the two test sessions: placebo and tyrosine.

An independent researcher randomized the order of tyrosine administration (tyrosine or placebo on the first test session) by means of a computer-generated order. The day before the test session from 10pm onwards, participants were asked to refrain from eating and drinking anything but water until arriving at the center the next morning, and to refrain from taking any medication that they would not take during both testing sessions, to avoid an unbalanced influence of this medication. The overnight fast prevents large variability in plasma LNAA levels between participants caused by the previous meal (Fernstrom et al., 1979). A similar fasting procedure has been adopted in other research using tyrosine supplementation (Lieberman and Wurtman, 1985; Banderet and Lieberman, 1989; Shurtleff et al., 1994; Mahoney et al., 2007; Colzato et al., 2013). Testing started from 8 or 10 A.M. at the latest and took ∼4.5 h (Fig. 2). The time of testing was kept similar for the placebo and tyrosine session of each participant (i.e., maximal difference between test sessions was 1 h, except for one participant for who the difference was 1.5 h).

The test day started with assessing subjective feelings of wellbeing measured with Bond & Lader visual-analog ratings. Blood pressure and heart rate were assessed, and a urine sample was provided. Next, participants started re-familiarization with all three tasks (always in the same order). The yoghurt mixture (with tyrosine or placebo) was provided such that participants entered the scanner 90 min after ingestion. Maximal concentration of plasma elevation and cognitive effects are seen approximately after 1.5 h and are normalized after 6–8 h (Glaeser et al., 1979). The stop-signal task (described below) was the first task performed in the scanner, followed by a working memory task. Scanning took ∼100 min. On exiting the scanner, a second urine sample was provided. After scanning, participants performed the third task measured effort discounting and completed a neuropsychological test battery assessing: immediate and delayed story recall (Wilson et al., 1985), digit span forward and backward (Wechsler, 1997), Stroop cards (Stroop, 1935), and verbal fluency (Tombaugh et al., 1999). Participants’ blood pressure, heart rate, and wellbeing were monitored three times during the test session.

### Experimental design: load-dependent stop-signal anticipation task

**Figure 3.**
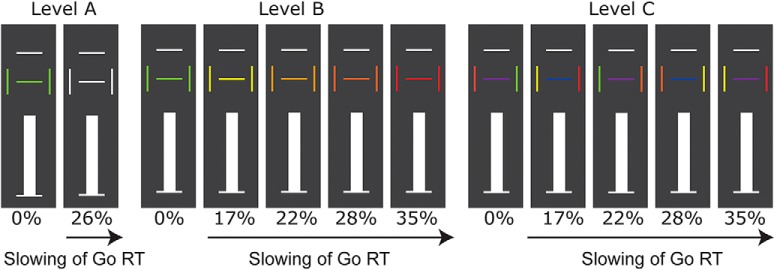
Load-dependent stop-signal anticipation task. Information load increased with level. Percentages reflect the probability a trial will be a Stop trial rather than a Go trial. For level B and C, stop-signal probability increased as a function of cue color. Every level contained 70 trials with 0% (green) and 270 trials with >0% (white in level A and various colors in levels B and C) stop-signal probability. Of these 270 >0% trials, 70 were Stop trials, with a mean stop-signal probability of 26%. For levels B and C, each >0% trial type contained 50 Go trials, plus a varying amount of Stop trials per color resulting in varying stop-signal probabilities (in between brackets): 10 yellow (17%), 14 amber (22%), 19 orange (28%), and 27 red (35%).

Participants performed a stop-signal anticipation task consisting of three levels differing in information load, which were presented in alternating blocks (Bloemendaal et al., 2016; Fig. 3). The paradigm consisted of Go trials and Stop trials. On every trial, a bar moved at a constant speed from a lower horizontal line toward an upper horizontal line, reaching a middle line (flanked by two vertical lines) in 800 ms. The Go task was to bring the bar to a halt as close to the middle line as possible, by pressing a button with the right index finger. A minority of trials were Stop trials. On Stop trials, the bar stopped moving automatically before reaching the middle line (the stop signal). This stop signal instructed the participants to withhold the planned Go response. The middle horizontal line and the two vertical lines represented cues that indicated stop-signal probability context by varying in color. To manipulate information load, the task consisted of three levels that were alternated in short blocks (Last alinea of Experimental design section). Between levels, stop-signal probability cues were varied in amount as well as in complexity. The stop-signal probability could be anticipated on the basis of the color of the cues (i.e., horizontal and vertical lines, presented 500 ms before the onset of the moving bar). Level A was the level with the least information load, with only white cues (stop probability of 26%) and green cues (stop probability of 0%). In level B, there were five types of Go trials with varying stop-signal probability, using an intuitive color range for the cues (Zandbelt and Vink, 2010): green, 0%; yellow, 17%; amber, 22%; orange, 28%; and red, 35%, with a mean of 26% stop probability. The non-green trials are collectively called >0% trials. Level C consisted of the same types and numbers of stop-signal probability cues as level B. However, in level C only one of the vertical lines signaled the correct stop-signal probability context. The correct side could be identified by the color of the middle line: a blue middle line indicated that the left vertical line color was valid, whereas a purple middle line indicated that the right vertical line color was valid.

We instructed participants that going and stopping were equally important and that it would not always be possible to suppress a response when a stop signal occurred. Participants were not informed of the exact stop-signal probabilities but were told that stop signals in all levels would not occur on trials with a green cue, and that stop signals in levels B and C were least likely in the context of a yellow cue and most likely in the context of a red cue, with the amber and orange cues coding intermediate, and, respectively, decreasing, stop-signal probabilities.

To ensure roughly equal numbers of successful and unsuccessful Stop trials, a staircase procedure adjusted stop-signal delay by 25 ms depending on stopping performance. Levels were presented in 34 blocks, each lasting 27 s and consisting of 10 trials, with an intertrial interval of 1000 ms. The sequence of trials and blocks were pseudo randomized (ensuring that the first three blocks of the task were always in order of levels A-B-C). Every level contained 70 trials with 0% (green) and 270 trials with >0% (white in level A and various colors in levels B and C) stop-signal probability. Of these 270 >0% trials, 70 were Stop trials, with a mean stop-signal probability of 26%. For levels B and C, each >0% trial type contained 50 Go trials, plus a varying amount of Stop trials per color resulting in varying stop-signal probabilities (in between brackets): 10 yellow (17%), 14 amber (22%), 19 orange (28%), and 27 red (35%). Two rest blocks of 20 s each were implemented at one-third and two-thirds of the task, respectively. The total task duration was ∼45 min. During the pre-screening, each level was explained and practiced separately for 48 trials (level A) and 72 trials (levels B and C). Participants were asked to repeat task instructions to ensure sufficient understanding. Then they practiced the task (levels were presented in alternating blocks) for 10 min. On each test day, instructions were rehearsed and the 10-min practice was repeated.

### Behavioral data analysis

#### Stop-signal task

All data were in accordance with the main assumptions of the race model (Logan and Cowan, 1984). Reactive response inhibition was measured by calculating the SSRT (stopping latency), according to the integration method (Verbruggen and Logan, 2009). Outliers on any outcome measure were determined using Grubbs’ test (i.e., they do not differ >2.8*SD from the mean; Grubbs, 1969). An ANOVA of SSRTs was used with the within-subjects factors level (A, B, C) and intervention (tyrosine, placebo).

RT slowing as a function of increasing stop-signal probability, indicated by the colored cues, indexed proactive response inhibition. Task non-compliance was determined by a negative difference on median RTs between 0% (green) and >0% (white) trials during the lowest cognitive load (level A). No participants were excluded based on this criterion. For each level, the slope of RTs was calculated as a function of stop-signal probability using a general linear model, resulting in a β value for the slowing slope. Hence, for level A, the slowing slope was calculated using the two proactive trial types [0% (green cues) and 26% (white)]. For levels B and C, the five proactive trial types were included in the slowing slope [0% (green cues), 17% (yellow), 22% (orange), 28% (amber) and 35% (red)]. Task instructions implied differential processing of 0% and >0% stop-signal probability trials, resulting in more slowing on >0% than 0% trials (i.e., a positive difference between these trial types). Level A consists of fewer cells than levels B and C and was therefore not compared with the other levels for the proactive inhibition analyses. An ANOVA was performed using the within-subjects factors level (levels B and C) and intervention (tyrosine, placebo). On lack of an interaction effect between level and intervention, the effect of intervention on slowing slope was assessed and reported on the average slowing slope irrespective of level. For effects of level independent of tyrosine manipulation, see Bloemendaal et al. (2016).

To assess the relation between age and tyrosine’s effects on reactive and proactive response inhibition, we added the covariate age in an ANOVA using factors intervention and level for proactive RT slowing and SSRT. On significant effects or interactions with intervention, we tested for a possible interaction between administration order and intervention. On lack of interaction effects with level, the effect of intervention and age was assessed irrespective of level.

### Neuropsychological measures and additional measures: catecholamine metabolites in urine, physiologic measures, and wellbeing

Outliers on any outcome measure were determined using Grubbs’ test (Grubbs, 1969), resulting in exclusion of one outlier on the HVA and one on DOPAC urine levels. Performance on neuropsychological tasks (digit span, verbal fluency, story recall, Stroop, box completion, letter cancellation) was assessed using paired t-tests comparing scores on the tyrosine session with the placebo session. The effect of tyrosine administration on catecholamine metabolites HVA, VMA, MOPEG, and DOPAC in urine was determined using four ANOVAs with within-subject factors time (T0, T2) and intervention (tyrosine, placebo). The effect of time on blood pressure, heart rate, and subjective wellbeing was assessed using ANOVAs with factor time (T20, T90, T240). The effect of tyrosine administration on these measures during the baseline corrected therapeutic window (T1 – T0) was assessed with a paired *t* test. Possible modulation of tyrosine’s effect on catecholamine metabolites, physiologic and neuropsychological measures by age was determined by adding the covariate age in an ANOVA on these measures using factors intervention (tyrosine, placebo). On significant effects, the influence of administration order was assessed in a separate ANOVA using within-subjects factor intervention (tyrosine, placebo), between-subjects factor administration order (tyrosine on first or second test day) and covariate age.

### MRI data acquisition and pre-processing

Whole-brain imaging was conducted on a Siemens TIM Trio 3T scanner (Magnetrom Skyra Tim, Siemens Medical Systems), using a 32-channel head coil. Functional data were obtained using a multi-echo gradient T2*-weighted echo-planar scanning sequence (Poser et al., 2006) with blood oxygen level-dependent (BOLD) contrast (34 axial-oblique slices, repetition time, 2070 ms; echo-times, 9.0, 19.3, 30.0, and 40.0 ms; in plane resolution, 3.5 × 3.5 mm; slice thickness, 3 mm; distance factor, 0.17; field of view, 224 mm; flip angle, 90°). Visual stimuli were projected on a screen and were viewed through a mirror attached to the head coil. In addition, a high-resolution T1-weighted magnetization-prepared rapid-acquisition gradient echo anatomic scan was obtained from each participant (192 sagittal slices; repetition time, 2.3 s; echo time, 3.03 ms; voxel size 1.0 × 1.0 × 1.0 mm; field of view 256 mm).

Preprocessing and mass-univariate data analysis were performed using SPM8 software (Statistical Parametric Mapping; Wellcome Trust Center for Cognitive Neuroimaging). Realignment parameters were estimated for the images acquired at the first echo-time and subsequently applied to images resulting from the three other echoes. The echo images were combined by weighting with a parallel-acquired inhomogeneity-desensitized algorithm, assessing the signal-to-noise ratio as described by Poser et al. (2006). Thirty volumes, acquired before the task, were used as input for this algorithm. After data quality check (i.e., for signal intensity spikes), the echo combined and realigned images were slice time corrected to the middle slice. The functional images were coregistered to the T1 scan. A sample-specific template was created by segmenting each individual T1 and using diffeomorphic anatomic registration to place each participant’s gray and white matter images in a study-specific space (Ashburner, 2007). Deformation parameters were stored in a subject-specific flow field. The coregistered fMRI images and anatomic T1 scan were nonlinearly normalized to the sample-specific anatomic template (using the subject-specific flow field), affine-aligned into a Montreal Neurologic Institute template, and finally smoothed using an 8.0-mm full width at half maximum Gaussian filter.

To exclude activation outside gray matter from second level analyses, GM normalized maps from all subjects in the sample were used to create an average gray matter mask, which was thresholded at a value of 0.4 (voxels with computed GM fractions >40% were selected, set after visual inspection) and applied as an explicit mask during second-level analysis.

### fMRI task analysis

The general linear model was set up as in (Bloemendaal et al., 2016), including twelve regressors of interest. For each level, we included four regressors: one modeling all Go trials (i.e., containing 0% and >0% stop-signal probability trials) and a corresponding parametric (i.e., proactive) regressor modeling stop-signal probability (six regressors: Go level A, Proactive level A, Go level B, Proactive level B, Go level C, Proactive level C). In level A, the parametric regressor consisted of two trial types. In level B and C, the parametric regressors consisted of five trial types. An actual stop-signal appeared on a proportion of >0% trials. These Stop trials were separately modeled as StopSuccess trials and StopFailure trials, based on whether or not the behavioral response was inhibited (six regressors: StopSuccess level A, StopFailure level A, StopSuccess level B, StopFailure level B, StopSuccess level C, StopFailure level C). As regressors of non-interest, we included a regressor for missed trials across all levels (i.e., no button box response on a Go trial), as well as a regressor modeling task instructions at the beginning of each mini-block. Moreover, twenty four realignment parameters were modeled as regressors of non-interest (six rigid-body movement parameters, plus Volterra expansion of these: first derivatives and quadratic derivatives of the original as well as first derivatives; Lund et al., 2005). Finally, to prevent contribution of global signal changes, we included signal from segmented out-of-brain voxels in the model as regressor of non-interest. All regressors of interest were modeled as delta functions at the onset of the trial and were convolved with a canonical hemodynamic response function. Time series were high-pass filtered (128-s cutoff) and serial correlations were corrected using an autoregressive (AR)1 model during classical (ReML) parameter estimation. Parameter estimates for the regressors of interest, derived from the mean least-squares fit of the model to the data, were used to estimate contrasts on the first level.

At the subject-specific, first level, we specified reactive and proactive contrasts within, across, and between levels. The first level contrast images were subsequently used in a second level random effects analysis to assess consistent effects across participants as well as the effects of intervention. Reactive response inhibition can be assessed using two different contrasts: StopSuccess > StopFailure or StopSuccess > Go. The contrast StopSuccess > StopFailure provides better control for stimulus-driven processing (i.e., presentation of the stop signal), and is orthogonal by design to the proactive inhibition contrast (which also involves the Go trials). The contrast StopSuccess > Go provides better control for the timing of the Go responses (i.e., Go and StopSuccess RTs are both slower than Stop Failure) and the outcome of the trial (i.e., both successful in Go and StopSuccess). We report effects on both types of contrasts. The parametric proactive regressors constituted the contrast for proactive response inhibition.

We assessed the main task effects of proactive and reactive response inhibition using the contrasts across levels and intervention. We explored level * intervention interactions within reactive and proactive response inhibition. On non-significant interactions, we assessed the effects of intervention across levels, comparing tyrosine and placebo sessions using a paired *t* test. On whole-brain corrected significant results, we assessed brain-behavior correlations in these clusters by extracting β weights using Marsbar (Tzourio-Mazoyer et al., 2002).

The sample-specific gray matter mask was applied as an explicit mask to the second-level statistics (see above). Statistical inference (pFWE < 0.05) was performed at the cluster level, correcting for multiple comparisons over the whole brain. The intensity threshold necessary to determine the cluster-level threshold was set at *p* < 0.001, uncorrected. On significant cluster-level activation, we assessed simple effects using subsequent ANOVAs or paired *t* tests.

## Results

### Behavioral results

Trait demographics and trait neuropsychological test scores are presented in Table 1. For a summary of statistical tests see Table 11.

#### Reactive response inhibition

##### Race model assumptions.

Data were in compliance with the main assumptions of the race model (Logan and Cowan, 1984). For each level and age group separately, mean response times (RTs) were faster on StopFailure versus >0% trials (paired *t* test, all *p* < 0.05) and mean RTs were faster for StopFailure RTs for short versus long SSDs (paired *t* test, all *p* < 0.05). Inhibition functions represent the probability of successfully inhibiting a response for every SSD, where the probability to inhibit decreases as the stop-signal is presented more closely to the moment that the response is made (Logan and Cowan, 1984). For each level, individual inhibition functions were calculated and displayed decreasing inhibition probability as a function of SSD.

**Figure 4.**
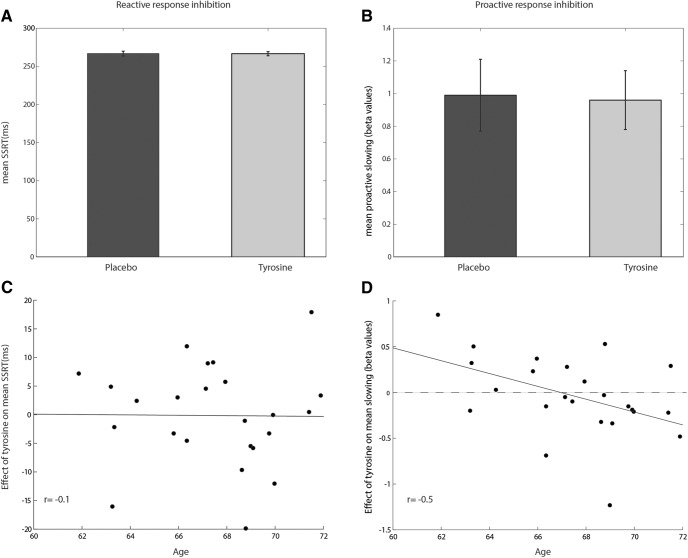
***A***, SSRT on placebo and tyrosine session across levels. ***B***, Proactive β slowing slopes on placebo and tyrosine session. Data represents mean, error bars represent SEM. ***C***, The effect of tyrosine compared with placebo on SSRT was not modulated by age (*r* = −0.1, *p* = 0.96). ***D***, With increasing age, tyrosine relative to placebo attenuated proactive RT slowing (*r* = −0.45, *p* = 0.03), i.e., the degree to which participants slowed their responses with increasing stop-signal probability_a,b_.

On non-significant tyrosine effects on SSRT between levels (*F*_(2,46)_ = 1.19, *p* = 0.31)_a1_, we performed our analyses across levels. Tyrosine administration did not affect SSRT across levels (*F*_(1,23)_ < 1)_a2_ and when assessing whether the effect of tyrosine administration depended on age, no effect was observed either (*F*_(1,22)_ < 1)_b1_ (Fig. 4*A*,*C*). Across intervention sessions, increasing age was associated with a slower SSRT (main effect of age: *F*_(1,22)_ = 4.3, *p* = 0.05, η^2^*_p_*= 0.16)_b2_.

#### Proactive response inhibition

On non-significant tyrosine effects on RT slowing between levels (*F*_(2,46)_ < 1)_c_, we performed our analyses across levels. Across levels and participants, tyrosine administration did not affect slowing β values (reflecting increasing slowing with increasing stop chance; *F*_(1,23)_ < 1)_d_. However, when adding age as a covariate, intervention did modulate proactive RT slowing (intervention * covariate age interaction: *F*_(1,22)_ = 5.7, *p* = 0.03, *r* = −0.5_e1_; main intervention: *F*_(1,22)_ = 5.6, *p* = 0.03, η^2^*_p_*= 0.20)_e2_ (Fig. 4*B*). Specifically, increasing age was related to a greater detrimental effect of tyrosine administration on RT slowing. Administration order did not interact with tyrosine administration on proactive slowing (*F*_(1,21)_ = 2.4, *p* = 0.14)_f_ (Fig. 4*D*).

In sum, the effect of tyrosine administration on behavioral measures of reactive response inhibition (i.e., SSRT) was not significant. However, age negatively modulated the effect of tyrosine on proactive response slowing: increasing age was associated with a greater detrimental effect of tyrosine on proactive slowing compared with placebo.

### fMRI results

#### Reactive and proactive inhibition activate frontoparietal networks and basal ganglia

**Figure 5.**
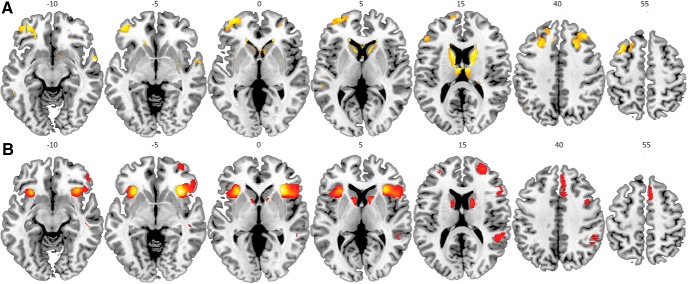
***A***, Main task effects across test sessions and level for reactive response inhibition – StopSuccess > fail. Images are thresholded at *p* < 0.001 uncorrected (for illustration purposes), cluster-level (pFWE < 0.05) significant clusters are listed in Table 2**_g_**. ***B***, Main task effects across test sessions and level for reactive response inhibition – StopSuccess > Go. Images are thresholded at *p* < 0.001 uncorrected (for illustration purposes), cluster-level (pFWE < 0.05) significant clusters are listed in Table 3_h_.

**Figure 6.**
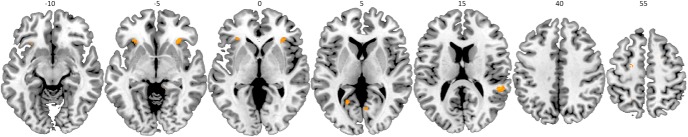
Main task effects across test sessions and level for proactive response inhibition (parametric regressors of Go). Images thresholded at *p* < 0.001 uncorrected (for illustration purposes), cluster-level (pFWE < 0.05) significant clusters are listed in Table 4_i_.

At our whole-brain corrected threshold of pFWE < 0.05 (cluster-level), main task effects revealed responses in a frontoparietal and striatal task network for reactive and proactive response inhibition (Figs. 5, 6; Tables 2–4)_g,h,i_ and deactivation of, e.g., motor cortex in the reactive response inhibition network, as shown previously for the current task in young and older adults (Bloemendaal et al., 2016) and for similar paradigms (Zandbelt and Vink, 2010; Kleerekooper et al., 2016).

**Table 2.** Whole-brain cluster-level significant task regions during reactive response inhibition across levels (StopSucces > StopFailure)_g_

Region	Peak MNI coordinates	pFWE value	Number of voxels in cluster
Right caudate	18 32 4	<0.001	1981
Left caudate	−10 15 10		
Left middle orbito-frontal gyrus	−38 62 2	<0.001	1258
Left middle frontal gyrus	−38 17 55	<0.001	1567
Left medial superior frontal gyrus	−18 30 46		
Right middle frontal gyrus	22 24 40	<0.001	894

**Table 3.** Whole-brain cluster-level significant task regions during reactive response inhibition across levels (StopSuccess > Go)_h_

Region	Peak MNI coordinates	pFWE value	No. voxels in cluster
Right insula	33 23 −3	<0.001	4104
Right inferior frontal operculum	51 18 7		
Left insula	−30 21 −5	<0.001	1472
Right caudate	14 11 10	0.007	379
Right supramarginal gyrus	52 −40 28	<0.001	1553
Right superior temporal gyrus	58 −42 19		
Right angular gyrus	52 −49 36		
Right supplementary motor area	8 17 48	<0.001	1498
Right medial superior frontal gyrus	8 42 34		
Right superior frontal gyrus	28 54 16	<0.001	891
Right middle frontal gyrus	26 51 24		
Left caudate	−10 11 6	0.017	314

**Table 4.** Whole-brain cluster-level significant task regions during proactive response inhibition across levels (parametric regressors of Go)_i_

Region	Peak MNI coordinates	pFWE value	Number of voxels in cluster
Right superior temporal gyrus	51 −37 12	0.011	248
Left insula	−28 26 −6	0.042	156
Left inferior frontal triangle	−32 32 1		

#### Tyrosine’s effects on reactive response inhibition (StopSuccess > StopFailure and StopSuccess > Go)

**Figure 7.**
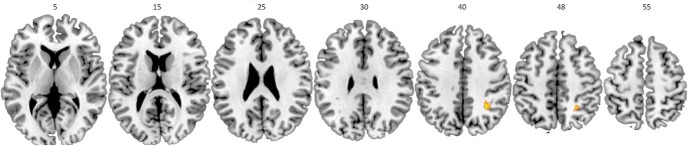
Positive correlation between age and effect of tyrosine on reactive response inhibition (StopSucces > StopFailure). Images are thresholded at cluster-level significant extent threshold (pFWE < 0.05; cluster-defining threshold: *p* < 0.001, uncorrected). AAL labels, *p* values, peak MNI coordinates, and number of voxels are listed in Table 5. The position of the slices is labeled with the *z* coordinates of the MNI atlas_l,m_.

In accordance with the behavioral results, no level * intervention interactions were observed during reactive response inhibition. Hence, effects of intervention are reported across level. Tyrosine did not affect neural signal during both contrasts of reactive inhibition (StopSuccess > StopFailure and StopSuccess > Go)_j,k_. A positive correlation between age and the effect of tyrosine on reactive response inhibition (StopSuccess > StopFailure) was observed in the right angular gyrus (Fig. 7; Table 5)_l_. With increasing age, tyrosine increased angular gyrus responses compared with placebo. None of these clusters demonstrated a brain-behavior correlation between tyrosine’s effect on reactive inhibition β values and SSRT. The reactive response inhibition contrast StopSuccess > Go did not yield a correlation with age_m_.

**Table 5.** Whole-brain cluster-level significant regions yielding a positive correlation between age and effect of tyrosine during reactive response inhibition (StopSuccess > StopFailure)_l,m_

Region	Peak MNI coordinates	pFWE value	Number of voxels in cluster
Right angular gyrus	34 −49 40	0.021	221

#### Tyrosine’s effects on proactive response inhibition (parametric regressor of Go)

In accordance with the behavioral results, no level * intervention interactions were observed during proactive response inhibition. Hence, effects of intervention are reported across level.

**Figure 8.**
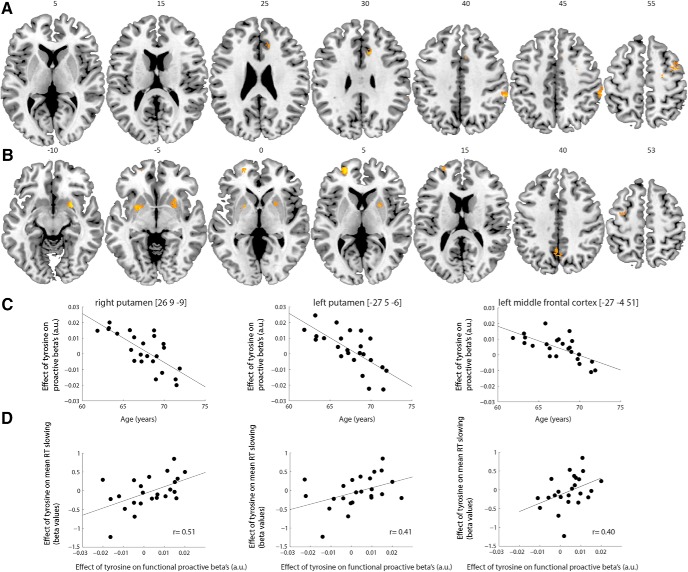
***A***, Effect of tyrosine versus placebo during proactive response inhibition (parametric proactive regressor). Images are thresholded at cluster level significant extent threshold (pFWE < 0.05). AAL labels, *p* values, peak MNI coordinates, and number of voxels are listed in Table 6. ***B***, Negative whole-brain correlation between age and effect of tyrosine on proactive response inhibition (parametric proactive regressor). Images are thresholded at cluster level significant extent threshold (pFWE < 0.05); cluster-defining threshold: *p* < 0.001, uncorrected). AAL labels, *p* values, peak MNI coordinates, and number of voxels are listed in Table 7. ***C***, For illustration purposes, the negative correlation between proactive βs and age is plotted for the regions showing a brain-behavior correlation (see below). ***D***, Regions with enhanced proactive signal after tyrosine with increasing age, correlated positively with tyrosine’s effect on behavioral RT slowing. The position of the slices is labeled with the *z* coordinates of the MNI atlas_n,o,p_.

Right middle cingulum, precentral and supramarginal gyrus signal increased after tyrosine administration compared with placebo (Fig. 8*A*; Table 6)_n_. We did not observe brain-behavior correlations between the effect of tyrosine on β values in these regions and the effect of tyrosine on proactive slowing. Concurrent with the behavioral results, age modulated the effect of tyrosine on neural signal during proactive response inhibition (parametric regressors; Fig. 8*B*; Table 7)_o_. With increasing age, tyrosine decreased signal in bilateral putamen, left middle and superior frontal gyrus, right supramarginal gyrus and left precuneus relative to placebo (depicted for illustration purposes in Fig. 8*C*)_._

**Table 6.** Whole-brain cluster-level significant regions for tyrosine versus placebo during proactive response inhibition (parametric regressors)_n_

Region	Peak MNI coordinates	pFWE value	Number of voxels in cluster
Right middle cingulum	12 20 31	0.006	229
Right anterior cingulum	12 32 21		
Right precentral gyrus	26 −12 54	0.006	233
Right middle frontal gyrus	40 0 55		
Right supramarginal gyrus	61 −34 42	0.014	197

**Table 7.** Whole-brain cluster-level significant regions yielding a negative correlation between age and effect of tyrosine during proactive response inhibition (parametric regressors)_o_

Region	Peak MNI coordinates	pFWE value	Number of voxels in cluster
Right putamen	26 9 −9	0.001	291
Left middle frontal gyrus	−27 −4 51	0.041	103
Left precentral gyrus	−34 −6 54		
Left superior frontal gyrus	−22 57 4	<0.001	356
Left middle frontal gyrus	−24 50 7		
Right supramarginal gyrus	62 −28 27	0.019	175
Left putamen	−27 5 −6	0.034	153
Left precuneus	−3 −61 42	0.049	141

Several of these regions (i.e., those with age-dependent tyrosine-induced decreases in proactive signal) correlated positively with tyrosine’s effect on behavioral RT slowing (Fig. 8*D*). Tyrosine-related decreases in fMRI signals were associated with tyrosine-related decreases in RT slowing in bilateral putamen (right: *r* = 0.51 *p* = 0.01; left: *r* = 0.41 *p* = 0.046), which was also marginally significant in left middle frontal gyrus (*r* = 0.40 *p* = 0.054)_p1,2,3_.

In sum, tyrosine increased right middle cingulum, precentral and supramarginal gyrus signal during proactive response inhibition. With increasing age, tyrosine decreased fronto-striatal and parietal proactive signal. Of these regions showing detrimental effects of tyrosine administration with increasing age, bilateral putamen (and left middle frontal gyrus) demonstrated a relation with tyrosine’s effect on behavior (i.e., proactive slowing). Moreover, with increasing age, tyrosine increased reactive signal in the angular gyrus. However, tyrosine effects in this reactive inhibition region did not show a relation with behavior.

### Effect of intervention on neuropsychological measures and additional measures: catecholamine metabolites in urine, physiologic measures, and wellbeing

Tyrosine administration did not affect neuropsychological measures (Table 8), nor was there an interaction between age and the effect of tyrosine administration on these measures.

**Table 8.** Effect of intervention on stop-signal task and neuropsychological tests_a,b_

Variable	Placebo	Tyrosine	*p* value	*p* value age
Story immediate recall (points)	9.5 (0.6)	10.2 (0.7)	0.3	0.68
Story delayed recall (points)	9.0 (0.6)	9.2 (0.6)	0.8	0.47
Digit span forward (points)	7.5 (0.4)	7.21 (0.4)	0.14	0.75
Digit span backward (points)	6.1 (0.4)	6.25 (0.5)	0.84	0.93
Stroop effect (s)	81.4 (11.1)	95.88 (12.5)	0.18	0.38
Stroop effect (errors)	.7 (0.2)	1.32 (0.3)	0.14	0.43
Verbal fluency DAT (items)	45.26 (2.3)	44.13 (2.3)	0.8	0.09
Block completion	92.23 (7.83)	83.42 (4.17)	0.36	0.84
Letter cancellation	249.29 (7.07)	250.17 (7.39)	0.61	0.35

Data represent mean (SEM). The first column with *p* values reflect the outcome of the paired *t* tests between tyrosine and placebo on the behavioral measure, the second column with *p* values reflects the interaction between tyrosine’s effect on the behavioral measure and age.

### Catecholamine metabolites in urine

At the beginning of the test day (T0) as well as approximately 3 h after ingestion of tyrosine or placebo (T2), a urine sample was provided by the participants. As expected when consuming yoghurt after a night’s fast, HVA, MOPEG, and VMA concentrations in urine were increased ∼3 h after ingestion of the mixture (T2) relative to the start of the test day (T0; main effects of time on HVA: *F*_(1,22)_ = 20.98, *p* < 0.001; MOPEG: *F*_(1,23)_ = 48.77, *p* < 0.001; VMA: *F*_(1,23)_ = 52.87, *p* < 0.001; Table 9)_q,r1,s_.

**Table 9.** Effect of tyrosine administration on catecholamine metabolites in urine_q,r,s,t,u,v,w_

Variable	Placebo		Tyrosine		
(mmol/M kr)	T0	T2	T0	T2	*p* value
HVA	2.80 (0.22)	3.36 (0.30)	2.74 (0.24)	3.20 (0.25)	0.43
MOPEG	1.08 (0.06)	1.34 (0.06)	1.14 (0.07)	1.3 (0.07)	0.097
VMA	1.75 (0.11)	2.1 (0.12)	1.75 (0.1)	2.0 (0.12)	0.006
DOPAC	1.21 (0.11)	1.25 (0.13)	1.10 (0.1)	1.28 (0.12)	0.013

Metabolite concentrations are expressed in mmol/mol creatinine, to correct for sample volume; *p* values reflect the interaction between drug and time from the ANOVA on tyrosine administration (tyrosine, placebo) and time (T0, T2). The effect of tyrosine on catecholamine metabolites did not interact with age.

Tyrosine administration affected catecholamine metabolites. VMA concentrations were lower after tyrosine compared with placebo (time * intervention interaction, *F*_(1,23)_ = 9.1, *p* = 0.006), driven by intervention differences in concentrations on T2 (*t*_(23)_ = 2.2, *p* = 0.036), not T0 (*t*_(23)_ = −0.078, *p* = 0.94)_r2_. However, DOPAC concentrations were higher after tyrosine compared with placebo (time * intervention interaction *F*_(1,22)_ = 8.8, *p* = 0.007), not driven by intervention effects on either T2 (*t*_(22)_ = −0.43, *p* = 0.67) or T0 (*t*_(23)_ = 1.3, *p* = 0.2)_t_.

Independent of intervention and time of measurement, VMA and DOPAC levels were modulated by age (main effect of age, VMA: *F*_(1,22)_ = 8.98, *p* = 0.007; DOPAC: *F*_(1,21)_ = 5.5, *p* = 0.024)_u,w_; increasing age was associated with higher urine levels of VMA and DOPAC. MOPEG and VMA levels interacted between time of measurement and age; increasing age was associated with a larger increase from time point T0 to T2 independent of tyrosine or placebo administration (MOPEG: *F*_(1,22)_ = 5.61, *p* = 0.027; VMA: *F*_(1,22)_ = 4.92, *p* = 0.037)_u2,v_.

### Wellbeing, blood pressure, and heart rate

Wellbeing ratings were assessed at the beginning of the test day (T0), at the assumed peak of tyrosine plasma level (T1) and at the end of the test day, ∼4 h after ingestion (T2). At the same time, systolic and diastolic blood pressure as well as heart rate were measured (Table 10).

**Table 10.** Wellbeing scales, blood pressure, and heart rate_x,y,z,aa_

Variable	Placebo			Tyrosine			*p* value
	T0	T1	T2	T0	T1	T2	
B&L total	8.1 (0.3)	7.7 (0.3)	8.1 (0.3)	8.0 (0.2)	7.6 (0.4)	8.0 (0.3)	0.97
B&L calmness	7.7 (0.4)	7.2 (0.5)	7.7 (0.4)	8.1 (0.3)	7.3 (0.5)	7.6 (0.4)	0.58
B&L contentness	8.4 (0.25)	7.6 (0.4)	8.5 (0.3)	8.3 (0.2)	7.6 (0.4)	8.3 (0.3)	0.99
B&L alertness	8.0 (0.3)	7.8 (0.4)	8.1 (0.3)	7.9 (0.3)	7.6 (0.3)	8.0 (0.3)	0.96
Systolic blood pressure	136.0 (3.8)	140. (4.0)	143.3 (3.8)	137.8 (3.8)	136.7 (3.5)	145.9 (4.4)	0.06
Diastolic blood pressure	84.0 (1.7)	80.8 (2.1)	85.71 (1.8)	82.8 (1.8)	80.0 (1.8)	87.2 (1.8)	0.72
Heart rate	63.4 (1.8)	63.6 (1.7)	61.2 (1.4)	63.4 (1.7)	63.2 (1.5)	62.7 (1.6)	0.72

B&L, Bond & Lader; *p* values reflect the outcome of the paired *t* tests between tyrosine and placebo on the baseline corrected physiologic measure (T1 – T0). No interactions between tyrosine administration and time or age were observed.

Over the course of the test day, changes in wellbeing as measured on the Bond & Lader total and contentness subscales were observed (main effect of time, total: *F*_(2,44)_ = 4.00, *p* = 0.025; contentness: *F*_(2,44)_ = 9.4, *p* < 0.001, respectively)_x,y_. Wellbeing decreased on T1 compared with T0 (total: *F*_(1,23)_ = 8.6, *p* = 0.008; contentness: *F*_(1,23)_ = 12.8, *p* = 0.002), and increased to baseline again on T2 compared with T1 and T0 (T2 vs T1 total: *F*_(1,23)_ = 4.5, *p* = 0.045 and contentness: *F*_(1,23)_ = 10.53, *p* = 0.004, T2 vs T0 total: *F*_(1,23)_ < 1 and contentness: *F*_(1,23)_ < 1). The subscales calmness and alertness did not change over time. The effect of tyrosine on the Bond & Lader subscale scores during the baseline corrected therapeutic window (T1 – T0) did not interact with age.

Systolic and diastolic blood pressure changed over the course of the test day (main effect of time systolic: *F*_(2,44)_ = 8.7, *p* = 0.001 and diastolic: *F*_(2,44)_ = 20.7, *p* < 0.001)_z,aa_. Diastolic pressure decreased on T1 compared with T0 (*F*_(1,23)_ = 18.1, *p* < 0.001), and both systolic and diastolic blood pressure increased on T2 compared with T1 (systolic: *F*_(1,22)_ = 8.4, *p* = 0.008 and diastolic: *F*_(1,23)_ = 27.6, *p* < 0.001). No effects of tyrosine administration or age were observed on systolic and diastolic blood pressure or heart rate during the baseline corrected therapeutic window (T1 – T0). Heart rate did not change over time.

The correlation between age and verbal IQ was non-significant (*r* = −0.221, *p* = 0.323). Verbal IQ was measured with the NART, which generally shows large cohort effects between age groups, with older adults performing better than younger adults (Uttl, 2002; see also unpublished observations from our lab (by M Bloemendaal, E Aarts, M van Holstein, R Cools) using the equivalent version in the native language of the participants). Therefore, this provides some confirmation of our hypothesis that there is minimal influence of generational differences on the current results with our small age range.

## Discussion

The current study investigated the neuro-cognitive effects of acute tyrosine administration, a dopamine precursor, on reactive and proactive response inhibition in a healthy older sample (aged 61–72 years; mean age: 67.5). Behaviorally, across the group, no effects of tyrosine administration on measures of reactive (i.e., SSRT) and proactive response inhibition (i.e., response slowing) were observed, although, neurally, proactive neural signal in right middle cingulum, precentral and supramarginal gyrus was increased by tyrosine. When taking age into account, age was found to negatively modulate the effect of tyrosine on proactive behavioral response slowing independent of cognitive load (i.e., level): increasing age was associated with a greater detrimental effect of tyrosine on proactive slowing compared with placebo. Functional imaging results were concomitant with the behavioral results: with increasing age, tyrosine decreased fronto-striatal and parietal proactive signal, but increased reactive signal in the angular gyrus. Brain-behavior correlations underline the behavioral relevance of modulated signal in these areas involved in proactive inhibition: tyrosine’s effects on bilateral putamen (and left middle frontal gyrus) signal correlated positively with tyrosine’s effect on proactive slowing. Such brain-behavior correlations were not observed for reactive inhibition.

The age-dependent detrimental effects of tyrosine on proactive slowing are surprising given that prior work has shown almost exclusively beneficial effects of tyrosine administration on cognition (for review, see Jongkees et al., 2015). Critically, these prior studies have all assessed young, not older adults. A study in adult schizophrenia patients (mean age 37.8 years, SD 6.8) displayed increased errors in a smooth pursuit saccades task in eight patients during three weeks supplementation of 10-g tyrosine daily (Deutsch et al., 1994). In line with an overdose hypothesis, a recent study demonstrated decreased working memory (i.e., n-back) performance with increasing tyrosine dose (from 100-150 to 200 mg/kg) in older adults (aged 60–75 years, mean age: 69.6; van de Rest et al., 2017). This cognitive overdose effect of tyrosine, which has so far been observed only in older adults, may be at least partly caused by a larger effective dose in older adults due to increased peripheral supply of tyrosine. Earlier research demonstrated increased plasma tyrosine levels in fasting older versus young women (Caballero et al., 1991) and increased plasma response in older versus young adults receiving the same dose (van de Rest et al., 2017). Critically, in this latter study, dose-dependent increases in plasma response correlated with dose-dependent decrements in working memory after tyrosine ingestion. Several peripheral processes can cause this presumed enhanced bioavailability; for example an age-related reduced first pass effect in the liver (Klotz, 2009), may result in higher amounts of tyrosine entering the blood stream in older adults. Furthermore, age-related insulin resistance (Caballero et al., 1991) may contribute to reduced peripheral amino acid uptake from the blood, resulting in higher amounts that reach the blood-brain barrier. The current results cannot identify the cause of enhanced bioavailability, which should be further studied. Results from the current study in urine metabolites support the idea of increased peripheral catecholamine precursor levels in older adults. Irrespective of intervention, we observed higher amounts of VMA and DOPAC across time and a higher increase of MOPEG and VMA metabolites with time, as a function of age. Thus, increased peripheral supply of tyrosine to the blood-brain barrier might have resulted in the currently observed age-dependent overdose effects on proactive response inhibition, despite the same oral dose per kilogram of bodyweight in every participant and despite increased dopamine deficits with aging.

Mechanistically, the conversion of tyrosine to L-dopa by the rate-limiting enzyme TH is inhibited by its final end products, i.e., the catecholamines dopamine and noradrenaline, present in the cytoplasm (Daubner et al., 2011). Indeed, a very high dose of phenylalanine, the conditional precursor of tyrosine, reduced dopamine release in the rat striatum, whereas lower doses increased dopamine release (During et al., 1988). The authors speculated that TH inhibition resulted in net reduced dopamine synthesis due to sudden high amounts of cytoplasmic catecholamines. Reduction of dopamine synthesis by inhibiting TH may also occur further in the dopamine signaling cascade, when an excess of dopamine increases dopamine D2 autoreceptor binding (Lindgren et al., 2001). The aging brain might be more sensitive to overshoots in auto-regulation, for example due to increased inflammatory markers, such as cytokines, which increase with age (Michaud et al., 2013) and can alter TH availability and auto-regulatory dopamine transporter expression (Felger and Miller, 2012).

Detrimental effects of tyrosine may be less surprising when considering literature on increased dopamine synthesis capacity in older adults, which is consistently observed when using the PET tracer FMT (Dejesus et al., 2001; Braskie et al., 2008; Berry et al., 2016), although mixed results have been obtained with another aromatic amino acid decarboxylase substrate, FDOPA, with decreased signal-to-noise (Martin et al., 1989; Sawle et al., 1990; Bhatt, 1991; Dreher et al., 2008). Increased age-related dopamine synthesis was negatively correlated with reward-related BOLD signal (Dreher et al., 2008) and, similarly, the positive relation between dopamine synthesis and cognitive performance seen in young adults was absent in older adults (Berry et al., 2016). We speculate that administrating extra precursor to a system with already high dopamine synthesis capacity may result in its inhibition.

The current results provide a first indication of age-related effects of tyrosine administration on dopamine processing. We used age as a continuous measure to assess its relation to tyrosine’s effects on neurocognition. However, looking at the scatter plots, it seems that the detrimental effects of tyrosine administration on proactive inhibitory responses in brain and behavior were especially apparent in the middle-old, sometimes referred to as old-old, group (70–79 years) relative to the young-old (60–69 years) participants. This is in accordance with the sub-group definition by some authors (Forman et al., 1992; de Almondes et al., 2016), although others have defined young-old individuals as 65-74 years old (Zizza et al., 2009; Moon et al., 2018) . In either definition, most of our participants were in the young-old group. However, age-related changes in the dopamine system have already been observed in similar age groups as in the current study, e.g., dopamine receptor binding differences in individuals up to 68 years old (Bäckman et al., 2000) and increased dopamine synthesis capacity in a group of older adults of on average 67 years old (Braskie et al., 2008). Nevertheless, previous dopamine findings were obtained by contrasting the effects between individuals with larger age differences than in the current study, although linear effects on striatal dopamine receptor binding with increasing age can be observed among the few participants that were in our age range of 61–72 years old (Wang et al., 1998). To strengthen our results, the peripheral and central mechanisms underlying age-dependent tyrosine effects on cognition should be investigated in future studies with larger age ranges and sample sizes, including measures of dopamine functioning. Moreover, the current results should be replicated using longitudinal designs, as cross-sectional designs cannot easily control for between-subject differences other than age, which could have contributed to the current results; even in this small age range.

Previous studies that have used 150 mg/kg, similar to the current study, have observed positive effects of tyrosine administration on cognition in young adults (Jongkees et al., 2015). However, it must be noted that these studies subjected participants to a stress intervention such as acoustic noise or a cold bath. One study did not use an external stress intervention other than the task at hand (Thomas et al., 1999) and only found positive effects of 150 mg/kg tyrosine administration on demanding multitasking. Stress or demanding circumstances increase neuronal firing and thereby catecholamine metabolism (Bliss et al., 1968), making these neurons more sensitive to precursor availability such as tyrosine (Scally et al., 1977). For this reason, a relatively high dose may be optimal in a high neuronal firing situation, but suboptimal during basal neuronal firing, even in young adults.

Effects of tyrosine administration were most prominent on behavioral and neural measures of proactive response inhibition. Behaviorally, with increasing age, tyrosine modulated only proactive response slowing, not SSRT. Neurally, tyrosine modulated signal in fronto-striatal and parietal regions during proactive inhibition, which was associated with its behavioral effects. Previous studies found evidence for dopaminergic modulation of proactive-like processes in response inhibition (such as post-error slowing and go accuracy; Bari et al., 2009; Bari and Robbins, 2013), without formally disentangling proactive and reactive response inhibition as in the current paradigm. Moreover, we show age-dependent effects of tyrosine on proactive response inhibition in, among others, bilateral putamen. The putamen was also found to be modulated by Go trial probability in a study by Dunovan et al. (2015), as well as in a network uniquely active during proactive response inhibition, as determined using independent component analysis (van Belle et al., 2014). These observations strengthen the interpretation that tyrosine has selectively modulated a functional network uniquely involved in processing proactive cues.

With noradrenaline being produced from dopamine, tyrosine administration could potentially also have contributed to increased noradrenaline synthesis. We cannot fully exclude this possibility, but given the presently observed tyrosine modulation of signal in the putamen, which is highly innervated by dopamine rather than noradrenaline (Nicola and Malenka, 1998), we hypothesize that the currently observed tyrosine effects are driven by dopaminergic neurons. Our hypothesis is strengthened by literature stating shortage of especially dopamine in the aging brain (Finch, 1973; Ota et al., 2006), rather than noradrenaline (Goldman-Rakic and Brown, 1981; Moretti et al., 1987).

During reactive response inhibition, tyrosine modulated angular gyrus signal with increasing age, which is only scarcely innervated by dopamine. Moreover, no behavioral effect of tyrosine on SSRT was observed, neither a correlation between tyrosine’s effect on this reactive region and SSRT. Therefore, the observed decrease in angular gyrus activation after tyrosine with increasing age might well reflect either an indirect result of tyrosine’s fronto-striatal effects on proactive inhibition or a noradrenergic effect in combination with a floor effect in SSRTs.

The majority of the catecholamine metabolites increased to a lesser extent after tyrosine compared with placebo administration (VMA significantly and MOPEG and HVA numerically). Only DOPAC levels increased after tyrosine compared with placebo. However, for unknown reasons, large baseline (T0) differences between intervention sessions were observed on this measure. This complicates the interpretation of the intervention effect on DOPAC levels. These mixed results should generally be interpreted with caution, as urine measures mostly reflect peripheral instead of central metabolites, with no clear link with central dopamine levels (Chekhonin et al., 2000).

In conclusion, we show age-related effects of tyrosine administration especially on proactive, not reactive, response inhibition, accompanied by signal changes in dopamine-rich fronto-striatal brain regions. Specifically, we observed that tyrosine’s effect on brain and cognition became detrimental with increasing age, questioning the cognitive enhancing potential of tyrosine in healthy aging.

Our results, particularly those in striatum, provide support for the hypothesis that proactive, but not reactive, response inhibition is modulated by dopamine.

**Table 11.** Summary of statistical analyses

Data structure	Type of test	Statistic	CI for the difference of intervention effect (unless otherwise specified)
Behavioral results			
Reactive response inhibition			
Interaction between intervention and level on reactive response inhibition (SSRT)	a1. RM ANOVA	*F*_(2,46)_ = 1.19, *p* = 0.31, η^2^*_*p*_* = 0.05	
Effect of intervention on reactive response inhibition (SSRT)	a2. RM ANOVA	*F*_(1,23)_ = 0.007, *p* = 0.93, η^2^*_*p*_* = 0.00	−3.87/3.57
Interaction between intervention and covariate age on reactive response inhibition (SSRT)	b1. RM ANOVA with coviarate age	*F*_(1,22)_ = 0.002, *p* = 0.95, η^2^*_*p*_* = 0.00	−3.961/3.66
Main effect of age on reactive response inhibition (SSRT)	b2. RM ANOVA with coviarate age	*F*_(1,22)_ = 4.3, *p* = 0.05, η^2^*_*p*_* = 0.16	
Proactive response inhibition			
Interaction between intervention and level (B,C) on proactive slowing βs	c. RM ANOVA	*F*_(1,23)_ = 0.11, *p* = 0.739, η^2^*_*p*_* = 0.01	
Effect of intervention on proactive slowing βs	d. RM ANOVA	*F*_(1,23)_ = 1.55, *p* = 0.68, η^2^*_*p*_* = 0.01	−0.22/.15
Interaction between intervention and covariate age on proactive slowing βs	e1. RM ANOVA with coviarate age	*F*_(1,22)_ = 5.7, *p* = 0.026, η^2^*_*p*_* = 0.21	−0.20/0.13
Main effect of intervention on proactive slowing βs	e2. RM ANOVA with coviarate age	*F*_(1,22)_ = 5.6, *p* = 0.027, η^2^*_*p*_* = 0.20	
Interaction between intervention effect and order on proactive slowing βs	f. RM ANOVA with coviarate age and order	*F*_(1,21)_ = 2.4, *p* = 0.136, η^2^*_*p*_* = 0.10	−0.20/0.13
fMRI results			
Reactive and proactive response inhibition task effects			
Task effects on reactive response inhibition across intervention and level (contrasts StopSuccess vs Failure and StopSuccess vs Go)	g, h. one sampled *t* test	Tables 2, 3	
Task effects on proactive response inhibition across intervention and level	i. one sampled *t* test	Table 4	
Intervention effects on reactive response inhibition (contrasts StopSuccess vs Failure and StopSuccess vs Go)			
Effect on intervention across levels on reactive response inhibition	j, k. paired *t* test	pFWE < 0.05, no significant clusters	
Interaction between intervention (difference between tyrosine and placebo) and covariate age on reactive response inhibition	l, m. one-way ANOVA with covariate age	For contrast StopSuccess vs Failure, see Table 5; contrast StopSuccess vs Go, no significant results at pFWE < 0.05	
Intervention effects on proactive response inhibition			
Effect on intervention on proactive response inhibition	n. paired *t* test	Table 6	
Interaction between intervention (difference between tyrosine and placebo) and covariate age on proactive response inhibition	o. one-way ANOVA with covariate age	Table 7	
Brain-behavior correlations between functional βs showing detrimental effects of tyrosine administration with increasing age and behavioral intervention effect (on proactive slowing βs)	p1. correlation	Left middle frontal gyrus (*r* = 0.40, *p* = 0.054)	0.01/0.70
	p2	Bilateral putamen (right: *r* = 0.51, *p* = 0.01)	0.13/0.76
	p3	Bilateral putamen (left: *r* = 0.41, *p* = 0.046)	0.01/0.70
Additional measures			
Catecholamine metabolites in urine			
Main effect of time on HVA	q. RM ANOVA	*F*_(1,22)_ = 20.98, *p* = 0.001	T0 vs T2: −0.52/0.20
Main effect of time on VMA	r1. RM ANOVA	*F*_(1,23)_ = 52.87, *p* = 0.000	T0 vs T2: −0.14/0.03
Time (T0, T2) * intervention interaction on VMA	r2. RM ANOVA	*F*_(1,23)_ = 9.1, *p* = 0.006; T2: *t*_(23)_ = 2.2, *p* = 0.036; T0: *t*_(23)_ = −0.078, *p* = 0.94	
main effect of time on MOPEG	s. RM ANOVA	*F*_(1,23)_ = 48.77, *p* = 0.000	T0 vs T2: −0.27/−0.15
Time (T0, T2) * intervention interaction on DOPAC	t. RM ANOVA	*F*_(1,22)_ = 8.8, *p* = 0.007; T2: *t*_(22)_ = −0.43, *p* = 0.67; T0: *t*_(23)_ = 1.3, *p* = 0.2	−0.24/0.010
Main effect of age on VMA	u1. RM ANOVA with covariate age	*F*_(1,22)_ = 8.98, *p* = 0.007	
Intervention * age interaction on VMA	u2. RM ANOVA with covariate age	*F*_(1,22)_ = 4.92, *p* = 0.037	1.68/2.07
Intervention * age interaction on MOPEG	v. RM ANOVA with covariate age	*F*_(1,22)_ = 5.61, *p* = 0.027	−0.06/0.08
Main effect of age on DOPAC	w. RM ANOVA with covariate age	*F*_(1,21)_ = 5.5, *p* = 0.024	
Wellbeing, blood pressure, and heart rate			
Main effect of time on B&L total	x. RM ANOVA	*F*_(2,44)_ = 4.00, *p* = 0.03; T1 vs T0: *F*_(1,23)_ = 8.6, *p* = 0.01; T2 vs T1: *F*_(1,23)_ = 4.5, *p* = 0.05; T2 vs T0 total: *F*_(1,23)_ < 1	T1 vs T0: −0.075/T2 vs T1: 0.82, −0.99/0.16; T2 vs T0: −0.44/0.37
Main effect of time on B&L contentness	y. RM ANOVA	*F*_(2,44)_ = 9.4, *p* < 0.001; T1 vs T0: *F*_(1,23)_ = 12.8, *p* = 0.002; T2 vs T1: *F*_(1,23)_ = 10.53, *p* = 0.004; T2 vs T0 total: *F*_(1,23)_ < 1	T1 vs T0: −0.08/0.82; T2 vs T1: −0.99/0.16; T2 vs T0: −0.44/0.37
Main effect of time on systolic blood pressure	z. RM ANOVA	*F*_(2,44)_ = 8.7, *p* = 0.001; T1 vs T0: *F*_(1,23)_ = 18.1, *p* < 0.001; T2 vs T1: *F*_(1,22)_ = 8.4, *p* = 0.008	T1 vs T0: −4.31/1.44; T2 vs T1: −10.77/−0.62
Main effect of time on diastolic blood pressure	aa. RM ANOVA	*F*_(2,44)_ = 20.7, *p* < 0.001; T2 vs T1: *F*_(1,23)_ = 27.6, *p* = 0.000	T2 vs T1: −8.56/−2.90

B&L, Bond & Lader.

## Synthesis

Reviewing Editor: Bradley Postle, University of Wisconsin Decisions are customarily a result of the Reviewing Editor and the peer reviewers coming together and discussing their recommendations until a consensus is reached. When revisions are invited, a fact-based synthesis statement explaining their decision and outlining what is needed to prepare a revision will be listed below. The following reviewer(s) agreed to reveal their identity: John Anderson, Youngbin Kwak

Here is the full text of the two reviews:

R1

Review of: Neuro-cognitive effects of acute tyrosine administration on reactive and proactive response inhibition in healthy older adults

Authors: -

Summary and overall impressions:

The authors conducted a within-subject double-blind placebo controlled experiment to examine the effect of tyrosine (a dopamine precursor), on two forms of inhibitory control, that is proactive, and reactive control. The authors report that while there are no main effects of tyrosine administration on performance, there is an interaction with age both behaviourally and neurally such that older adults (within the older adult sample) show performance decrements on proactive slowing, and decreased fronto-striatal and parietal signal.

I can appreciate how difficult it must have been to collect these data, within subject designs with a drug manipulation aren't easy to get past ethics boards, nor is it easy to get participants. Notwithstanding the value of the data, there are some issues with language, the fMRI results, and careful editing (perhaps employ a proof-reader) which will need to be addressed before this manuscript can be published. I feel certain the authors are able to manage the changes I recommend.

Caveat emptor: my expertise is the cognitive neuroscience of aging, statistics, and fMRI analysis - I have no background in neurochemistry & have not commented on the accuracy of those sections (though I see the two earlier reviews do).

Recommendation: Accept with major revisions.

Section by section breakdown:

Introduction:

The introduction is generally well written and clear. Citations to Lynn Hasher & Adam Gazzaley should be inserted at line 56 to help contextualize inhibition in aging. Line 74 contains a typo (young should be younger).

The authors may wish to consider framing their findings in terms of the existing literature on ‘young-old’ and ‘oldest-old’ individual, though they should bear in mind that individuals categorized as ‘oldest-old’ are 70-80 years (most of the author's participants fall in the ‘young-old’ category).

Lines 76-78 are extremely unsatisfactory - the authors make a statement that “using a smaller age rage (typo - should be range), we do not expect generational differences to influence the effects of dopaminergic agents, such as differences in education or computer experience that would differ in a cross-sectional design with larger age differences.”

Did the authors actually test this? If so, they should report this non-significant difference.

The paper by Van de Rest 2017 does not appear in the citation list despite featuring prominently in the introduction and the discussion. Please add.

While I understand that the previous reviewer argued that the ‘lack’ of a strong hypothesis about age-related negative effects was a fault with the paper, inserting a hypothesis post-hoc is terrible science. Please undo this edit and move any discussion of negative effects of tyrosine (which is obviously post-hoc) to the discussion. It seems to me that you started with a relatively exploratory design with the hopes of finding a positive effect of tyrosine in older adults (mirroring the effects in younger adults). Not finding this effect is interesting, and it should be framed as such. Please make it clear that you hypothesized a beneficial outcome (if indeed this was the case), and the hypothesis was disconfirmed.

Method & Results:

As a first step, I ran stat-checker on the PDF file & all the results are accurate as reported :).

I next took a look at your participants section. I found it quite difficult to follow which participants were excluded. Please clarify this section, it might be easier to follow if it was organized as: “we recruited 33 participants for the study. Of the 29, 3 were excluded for ..., 2 did not complete the stop-task, and 3 others were excluded for excessive movement... This left a final sample of 24 subjects for analysis.”

The rationale for the dosage 150 mg/kg of tyrosine per participant only becomes clear in the discussion when the authors make it clear that this is the typical dosage for younger adults. At this point, it also becomes clear that using this dosage, younger adults typically have positive outcomes. Please move this logic earlier in the paper otherwise your poor reader is left wondering how you decided on the dosage.

The wording in lines 152-153 is strange. In English this sounds backwards to what I think you mean. You write “ All subjects were tested at a location that will be identified if the article is accepted between November 2014 and August 2015.” Which makes it sound as if you will only reveal the location that participants were collected from if the article was accepted in the past - between 2014 and 2015. You could re-write this as: “Data were collected between 2014 and 2015. We will reveal the location the data were collected upon publication.” Unless you have a time-machine...

Lines 158-159, ‘weighted’ should be ‘weighed’ - also, this is standard procedure & not necessary to include (all MRI techs will weigh participants to adjust RF signal to prevent overheating).

Is it possible that there are order effects associated with the fixed presentation of the stop-signal task before the working memory task? Participant fatigue (particularly since they were fasting) is likely to build up across time & may make the working memory task systematically poorly performed.

Please put more information in Figure 3's caption. I am one of those people who attempts to read the abstract, skip to the figures and tables and skim the discussion for a sense of the paper before reading it more thoroughly. Figure 3 is only interpretable by reading the text. Try to ensure that someone could interpret the figure and caption on its own.

The MRI preprocessing is ok, but your results look very noisy, so let's try to improve this. In figure 5A for instance, I see ‘activation’ in the white matter of the corpus callosum and the anterior ventricles. This is also apparent in Figure 6 (activation in the genu of the corpus callosum), and Figure 7. In, Figure 7 most of the activation is white matter. What you're calling calcarine gyrus and bilateral lingual gyrus does not appear to be in grey matter at all. I suggest the following:

Consider eroding the white matter & CSF masks by 1 voxel & adding them as regressors. If you don't erode these images, you'll capture grey matter BOLD signal and end up with messier results. I highly recommend this step, it's quite similar to what ANATICOR in AFNI does to clean data.

Consider using independent components analysis (ICA) either with GIFT or MELODIC to identify residual noise in your dataset as a last step before analysis.

Potentially the easiest thing to do would be to simply use every participant's grey matter image to mask their data & exclude white matter and CSF from the analysis.

Just out of curiosity, do your participants have a large number of white matter hyperintensities? These can sometimes (falsely) manifest as BOLD signal in white matter regions. If so, this might help to explain the weird white matter results.

I'm not sure what all the footnotes after every set of statistical results refer to - please explain.

On page 18, line 377, it's not necessary to report both a partial eta square value and an r value (both are effect sizes). I assume this is related to the power analysis discussed on page 25? More on that in a bit...

I notice that you report that systolic and diastolic blood pressure changed over the course of the test day. Please consider reporting when your participants were tested and whether they were all tested at the same time (e.g. 9:00 AM, or not). If so, some of the effects may be due to time of day - older adults are known to have their circadian peak in the morning, and this coincides with differences in fMRI activations and connectivity.

Discussion:

The discussion is well written. That said, I fail to see the significance of a post-hoc power analysis (no pun intended). Post-hoc power analyses are not meaningful & should not be interpreted. After you've collected the data and run the statistics, either you detect the effect (in which case, yay, you had enough power to detect the effects), or you don't.

Well done, good luck, nice paper.

John A.E. Anderson

R2

R2

Line 57: More details on reactive inhibition and proactive inhibition required. Specifically, what is proactive inhibition under high information load? 

Line 71-76: As the authors say, previous studies comparing older and younger adults show significant differences in dopaminergic activity. However, is there evidence to believe that within the narrow age range of older adults (for example 61-72 yrs tested in your study) the endogenous levels dopaminergic activity linearly varies with age? The authors mentioned in line 79 that ‘presumably’ the oldest old is more deprived with dopamine, and the results showing greater detrimental effect of tyrosine in older of the older adults is interpreted with only an assumption that the older folks are more deprived of dopamine. It would be great if there were some scientific evidence for this. 

256: The analysis not including Age as a covariate did not include Level A (only B and C was included), but in the analysis with Age as a covariate did include Level A. Why was this done so? 

364-368: what do the a1-b2 labels right next to the stats value mean? In fact I've noticed these labels throughout the result section. 

Figure 4 C and D: It would be helpful to include correlation r values next to the regression line in the graph. 

454-459: The effect of age on concentration of VMA, DOPAC and MOPEG, may be of critical importance in explain the data. Are any of these measures known to be associated with endogenous levels of dopaminergic activity? As I've mentioned earlier, the entire story of the manuscripts is built around the idea that with increasing age, there's lower dopaminergic activity as well as an up-regulation of dopamine-synthesis capacity. However there's no evidence from the collected data that tell this is true. Would any of these Catecholamine measures help address this issue? If this is the case, then can you include these measures as covariates in the behavioral and fMRI analysis to determine if the effect of age on Tyrosine's effect on proactive slowing is mediated by age-related differences on dopaminergic transmission? In fact if there isn't such effort to relate age-related difference in Tyrosine's effect to some aspect of real dopaminergic functioning, we can't be sure if this is indeed mediated by age-related changes in dopaminergic functioning. It may well be that the older people are just worse after taking any meds, which may not be specific to dopaminergic functioning. 

The authors may argue that this effect was selectively shown on pro-active but not in reactive inhibition, which could provide some specificity of the effect. But then why for pro-active and not for re-active inhibition? This could partly be explained by reviewing the distinctive neural circuitries know to be involved in each process. Much of Adam Aron's work as well as a recent work by Tim Verstynen (Dunovan et al., 2015, eLife) suggest distinctive neural processes for the two inhibitions. In their data, they have shown that the networks involved in each process are vastly overlapping in the fronto-parietal regions, yet they see different results for each in age related tyrosine effect. Is one (pro-active) more dependent on striatal regions than other (reactive), which may potentially explain the difference in age-related effects? 
